# A Review on Blood Reference Values as a Valuable Marker of Wildlife Welfare in *Erinaceus europaeus*

**DOI:** 10.3390/ani14060982

**Published:** 2024-03-21

**Authors:** Sofia Rosa, Ana C. Silvestre-Ferreira, Felisbina Luísa Queiroga

**Affiliations:** 1 Animal and Veterinary Science Research Centre (CECAV), University of Trás-os-Montes and Alto Douro (UTAD), 5001-801 Vila Real, Portugal; sofiaasrosa99@gmail.com (S.R.); aferreir@utad.pt (A.C.S.-F.); 2 Department of Veterinary Science, University of Trás-os-Montes and Alto Douro (UTAD), 5001-801 Vila Real, Portugal; 3 Associated Laboratory for Animal and Veterinary Science (AL4AnimalS), 1300-477 Lisboa, Portugal; 4 Center for the Study of Animal Sciences (CECA-ICETA), University of Porto, 4099-002 Porto, Portugal

**Keywords:** western-European hedgehog, hematology, biochemistry, reference intervals, One Health, animals’ health

## Abstract

**Simple Summary:**

This review addresses the health challenges of western-European hedgehogs in human environments, emphasizing their exposure to contaminants and diseases. As vital contributors to One Health studies, hedgehogs serve as indicators of environmental health. This study underscores the importance of comprehending their well-being via blood value exploration, which is crucial for identifying threats and ensuring their conservation. By analyzing the existing literature on hedgehog blood reference values, this review emphasizes the ongoing necessity for research to create a valuable tool for the assessment of species health.

**Abstract:**

The western-European hedgehog (*Erinaceus europaeus*), in expanding its range towards human habitats, faces exposure to contaminants and biological agents, potentially leading to diseases associated with hematological and biochemical changes. As bioindicators of environmental pollution and carriers of zoonotic agents, hedgehogs play a crucial role in One Health studies, emphasizing the need for a comprehensive understanding of their clinical-pathological aspects. Exploring the blood reference values in healthy animals of this species is crucial for understanding and improving their well-being, and identifying possible diseases/pathogens that may affect its conservation and/or impact human health. This review is focused on analyzing the data available in the literature for *Erinaceus europaeus* blood reference intervals. A comprehensive literature review of the studies published in Europe is performed, highlighting their specificities, and emphasizing the need for continuous research in this field. Our final goal is to provide a crucial tool for assessing the health status of the species, and underscoring the significance of research in this specific domain.

## 1. Introduction

### 1.1. Erinaceus Europaeus: From Health to Disease

The western-European hedgehog, *Erinaceus europaeus*, belongs to the mammal order Eulipotyphla [1], family Erinaceidae, subfamily Erinaceinae, and genera *Erinaceus* [2]. There are four distinct hedgehog species in Europe: *Erinaceus europaeus*, *Erinaceus roumanicus*, *Erinaceus concolor*, and *Atelerix algirus*. *E. europaeus* is distributed in western and central Europe, including regions such as Britain, the Mediterranean Islands, southern Scandinavia, Estonia, and northern Russia [3,4].

Known for its adaptability, *E. europaeus* thrives in various environmental conditions. For example, it is the prevalent species in Portugal, with a higher incidence in the southern regions; however, the data suggest a broad distribution, extending into the central and northern areas of the country [2,5].

Exhibiting plesiomorphic characteristics in their morphology, physiology, and behavior, hedgehogs are considered highly primitive mammals. As nocturnal, terrestrial insectivores, hedgehogs rely predominantly on their acute senses of hearing and smell. Their anatomy includes brown and white cutaneous spines, vital for defense against predators, projecting spines in all directions, which forms a complete body envelope [4,6]. Typically solitary, they interact socially during breeding or foraging [1,7].

Hedgehogs are found in landscapes with ecotones formed by shrubs and hedges, often in rural or semi-urban habitats. However, their natural habitat can include forests, grasslands, scrublands, or cultivated areas. Their preference leans towards wetlands in regions influenced by the Atlantic, and forested regions and/or mountainous and humid terrains in areas with a Mediterranean influence [5]. 

Their diet consists of insects, snails, slugs, earthworms, and small vertebrates, with beetles being a primary energy source. Anthropogenic pressures, like extended urbanization areas, have led to a dietary shift, including fish, meat, milk, and even pet food. These dietary adaptations showcase the hedgehog’s remarkable ability to adjust to changing environments [8,9]. 

These animals hibernate in response to low temperatures (<8 °C) and photoperiods, reducing activity, temperature, respiration, and metabolism from September to May. Before they hibernate, hedgehogs meticulously construct a specialized and sturdy nest, known as a hibernaculum, where they seek refuge during the hibernation period. Throughout this time, their body temperature drops, and respiratory and heart rates slow considerably [1,7].

Reproduction season is tied to hibernation duration and geographic location, with sexual behavior initiating soon after hibernation, and intensifying between May and July, then persisting until August. This polygamous species allows both males and females to engage in multiple matings during the breeding season. The gestation period spans from 31 to 39 days, culminating in the birth of hoglets after approximately 5 weeks [1,10].

The species *Erinaceus europaeus* is on the International Union for the Conservation of Nature (IUCN) red list, denoting “least concern” due to their high population levels [11]. However, in recent decades, there are reports of a decrease in the number of individuals of this species. This decline can be attributed to various factors, including natural mortality during hibernation, the constant threat of predation from badgers and others, and the occurrence of road traffic accidents, even though most avoid travelling upon roadways [12]. Furthermore, the situation is exacerbated by the effects of habitat alteration, climate change, exposure to pesticides, and soil contamination. Together, these elements significantly undermine their survival dynamics [13,14]. 

Furthermore, the complexities of their existence unfold as hedgehogs harbor a diverse array of parasites and pathogens, thereby intensifying the challenges they face. This contributes significantly to the augmented mortality rates and decreased reproductive success. Some examples of ectoparasites for this species are fleas (*Siphonaptera*) that are usually found on the front legs, the neck, the head, the chest, and the belly of the host; and mites and ticks (Acari), which usually prefer to inhabit the host’s whole body, especially the furred body sections, the areas around the eyes, ears, and the region around the anus. These animals also host endoparasites such as nematodes like *Crenosoma striatum*, which is the most prominent parasite of the lung; trematodes; cestodes (not very common); and acanthocephalans. They also host bacteria, viruses, fungi, and protozoa [1,12,15,16] (Table 1).

The western-European hedgehog, distinguished by its intricate ecological habits and multifaceted interactions with various animals and humans, unequivocally emerges as a pivotal sentinel in a comprehensive One Health approach, offering invaluable insights into the intricate ecology of emerging viruses. These fascinating creatures not only serve as bioindicators for discerning environmental contamination, but also play a crucial role as the host for a diverse array of tickborne zoonotic agents, thereby presenting a myriad of potential threats upon both direct and indirect physical contact.

Research has identified hedgehogs as indicators for antibiotic resistance, specifically showing the presence of resistance genes to tetracyclines. Additionally, they are recognized as hosts for methicillin-resistant *Staphylococcus aureus* (MRSA) [17,18].

Systematic reviews highlight *E. europaeus* as carriers of zoonotic pathogens, with urban areas posing a higher risk of interaction between hedgehogs and humans or companion animals, thus underscoring the importance of balanced coexistence and responsible interactions. This urban interaction may have both positive aspects, such as aiding in pest control, and negative consequences, including the potential transmission of diseases and the risk of injuries to hedgehogs and companion animals. In essence, the ecological role of hedgehogs extends beyond their charming appearance, making them crucial indicators and potential carriers of infectious agents in our shared environment.

The western-European hedgehog, serving as a crucial biomarker for the collective health of animals, humans, and the environment, provides valuable insights into environmental contaminants, zoonotic agents, and emerging diseases. Their role in early detection and monitoring is pivotal, and the establishment of reference intervals becomes essential, acting as a reliable benchmark in the assessment of hedgehog health and the identification of potential broader health issues. Essentially, hedgehogs, as biomarkers, illuminate the intricate connections between species and act as sentinels, reflecting the overall health of ecosystems [19,20].

### 1.2. The Value of the “Normal”

Blood reference intervals (RIs), also known as reference ranges or normal ranges, represent the range of values for a particular physiological or laboratory parameter within a healthy population. These intervals are established based on data collected from a group of healthy individuals who are in similar environmental conditions and have undergone the same or similar analytical methods for testing. Reference intervals stand as the predominant medical decision-making tool, forming the basis of laboratory testing, and holding a substantial role in the interpretation of results, complementing result quality [21]. Two methods, direct and indirect, are employed for RI determination, with the former involving samples from a pre-selected reference population, and the latter relying on routine test results [22]. 

While published RIs are available, their appropriateness can vary, emphasizing the necessity for laboratory-specific intervals, considering population, instrumentation, and reagent variations [23,24]. The RI study, following the Clinical and Laboratory Standards Institute (CLSI) and the International Federation of Clinical Chemistry (IFCC) recommendations, serves as a crucial data source [21]. The American Society of Veterinary Clinical Pathology (ASVCP) guidelines further emphasize the need to define the population of interest and confirm the health status of selected individuals for RI determination [23]. During an RI study, it is crucial to ensure that the subjects in the reference population closely resemble the group for which the test will be applied. To ensure accurate RI studies and result interpretation, pre-analytical factors must also be considered, including sample type and handling. Some tests may differ due to age, gender, breed, or environmental factors [21], as previously observed in the western-European hedgehog [7,25,26]. 

**Figure 1:**
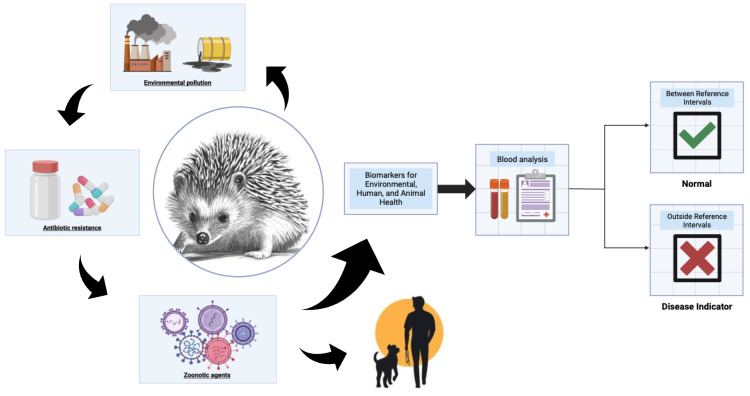
One Health approach for hedgehogs, emphasizing the significance of blood analysis and the establishment of RIs for assessing hedgehog health and welfare.

Determining RIs for the routine hematological and biochemical parameters in wild, healthy animals is a crucial initial step for identifying potentially ill individuals. This approach gains significance in *E. europaeus* because specific zoonotic agents, such as bacteria or viruses, may adversely affect various internal organs, including the liver, kidneys, or respiratory system, and may also be potentially transmissible to humans. These impacts can be precisely detected through alterations in hematological and biochemical parameters, such as blood cell counts or liver and kidney function markers. Recognizing these alterations in hedgehogs not only helps in identifying at-risk populations, but also highlights the importance of this study within the One Health framework [27] (Figure 1).

## 2. Hematologic and Serum Biochemistry Reference Intervals for *E. europaeus*: Where Are We?

Establishing hematologic and serum biochemistry reference intervals is crucial for understanding the health status of animal populations in the wild [27]. Research on the *E. europaeus* is currently limited, with only three studies focused on determining RIs for hematological parameters and two studies investigating RIs for biochemical analysis. All of these studies have been conducted on healthy animals housed in recovery centers, facilitating sample collection from a sufficient number of individuals. However, it is important to note that findings from these studies may not entirely reflect observations in natural habitats. Next, we will briefly describe the main findings from these studies.

In the study performed in England and published in 2002 by Lewis et al. [25], 50 hedgehogs, comprising 29 males and 21 females, all over six months of age, were examined. They had been overwintered at the Wildlife Hospital Trust in Haddenham, Buckinghamshire. The hedgehogs were kept outdoors in small groups, and were fed a combination of tinned and dry proprietary dog food, with access to water ad libitum. Upon arrival at the hospital, they were diagnosed with lungworm infections, ectoparasites, and other medical conditions, all of which were subsequently treated. The blood samples were taken in March (Spring), and, throughout that process, 0.5 mL of blood was drawn from the medial saphenous vein with EDTA, under anesthesia using isoflurane and oxygen administered via a coaxial circuit and facemask. For the first time, this study provided values for hemograms of healthy *E. europaeus* in captivity*,* but lacks information on biochemical and protein electrophoresis parameters (Table 2). 

In the study by Rossi and colleagues [28], published in 2014, hedgehogs from three rehabilitation centers in Northern Italy were investigated. Blood was collected from 50 hedgehogs, likely juveniles with unknown gender distribution, between March and June (spring). Sampling, under general anesthesia, used a mix of 2% isoflurane and oxygen applied via a facemask; the samples were then drawn from the jugular vein and placed in EDTA tubes. Some hedgehogs were captured in autumn and housed in two centers, while others were born in the third center. Housing included groups of four or five animals, or solitary arrangements for easier treatment administration. All centers provided a daily diet of 20–30 g of a mix of dry and wet commercial cat food, supplemented with apple slices and ad libitum water at varying intervals. This study explores the impact of overwintering on hedgehog hematologic (Table 2) and biochemical variables, describing, for the first time, a set of routine serum biochemical parameters for this species (Table 3). 

In the study carried out in northern Portugal [26] by Rosa and colleagues, published in 2023, data from 37 healthy hedgehogs kept in captivity at the Wild Animal Rehabilitation Center of UTAD, Northern Portugal were analyzed. The gender distribution among the records revealed thirteen males and twenty-two females, with information missing in two cases. Regarding age, details were available for 34 out of 37 cases, indicating that 20 were juveniles, 14 were adults, and information for the remaining cases could not be obtained. During the study, animals were anesthetized using a mask administration of isoflurane, and, following a loss of reflexes, the isoflurane concentration was reduced and maintained during the physical exam and sample collection. While the animal was under anesthesia, blood samples were obtained via a cranial vena cava puncture, and were then transferred into 0.5 mL lithium heparin tubes. Although this research does not specify the time of harvest, it encompasses a broader scope, including hematology, biochemistry, and protein electrophoresis reference intervals, thus offering unique data that has not been covered in the previous studies (parameters such as RDW, MPV, and PCT for hematology; AST, total bilirubin, sodium, potassium and chloride for biochemistry and the electrophoretic profile) (see Table 2 and Table 3). 

## 3. Discussion

The western-European hedgehog (*Erinaceus europaeus*) is an insectivorous mammal, widely distributed across Europe. In urban environments, hedgehogs often coexist closely with humans, potentially exposing them to various contaminants and biological agents [28] which may impact their health. Furthermore, as they also carry zoonotic agents, close coexistence and shared habitats can also be dangerous for humans [29].

Blood reference intervals for wild healthy animals are vital tools in both veterinary medicine and wildlife conservation. They establish a crucial baseline for assessing the health status of individuals within a population, allowing for the identification of deviations that may signal underlying health issues or exposure to environmental stressors. In wild animal populations, exposure to contaminants and/or infectious agents is a common occurrence, particularly in areas where human activities overlap with natural habitats. Urban areas pose significant challenges for wildlife health, as animals sharing these environments are frequently exposed to pollutants, pesticides, and other human-induced threats. The regular monitoring of hematological and biochemical parameters in wild animal populations enables the early detection of health disturbances, empowering the implementation of proactive measures to mitigate potential threats [30,31]. 

An essential aspect of this monitoring involves detecting heavy metal exposure, given the potentially harmful effects on both wildlife and human health. Heavy metals like lead, mercury, zinc, and cadmium are commonly present in the environment due to industrial activities, mining, and urbanization. These metals have the capacity to accumulate in animal tissues and organs, leading to a range of health issues, including liver and kidney damage. Consequently, measuring the biochemical parameters associated with heavy metal exposure, such as alanine aminotransferase (ALT) and creatinine, offers valuable insights into the health status of wildlife populations. Such data can inform conservation strategies aimed at mitigating the impacts of environmental contamination on both animal and human health [32].

Table 2 and Table 3 provide a comprehensive overview of published RI values for key blood parameters, therefore enhancing our understanding of the hematologic and serum biochemical profiles of healthy western-European hedgehogs [25,26,28]. One aspect we want to highlight is that the existing published data are solely derived from animals kept in captivity, prompting careful consideration when applying these findings to wild populations. In fact, it is important to account for the stress experienced in captivity and during handling when interpreting the results of hedgehogs under investigation. Such stressors could potentially influence outcomes, thereby impacting the reference intervals [29]. Another aspect to consider are the slight variations observed between studies conducted in different countries across Europe. These subtle differences, likely influenced by the geographic distribution of the studies, emphasize the necessity for more extensive research across the continent. This aspect underscores the importance of tailored RI determinations in conservation and rehabilitation practices within diverse geographic locations. Such an approach enhances individualized interpretation, leading to improved diagnostic accuracy. By integrating these findings, we can reach a more holistic understanding of *E. europaeus*, thereby making significant contributions to species conservation [33]. Additionally, variations in methodologies and laboratory practices may introduce differences that could potentially impact the results [34].

## 4. Conclusions and Future Directions

This review underscores the scarcity of RIs for the western-European hedgehog, highlighting the critical need for ongoing investigations. Reference intervals are crucial for identifying unhealthy individuals, therefore contributing to species conservation and global biodiversity. The limited data available in the literature underscore the significance of ongoing investigations, aiding in species characterization, as well as facilitating the early diagnosis of diseases. 

In future investigations of *Erinaceus europaeus*, the exploration of hematological and biochemical values could delve into seasonal variations, conduct longitudinal studies to monitor age-related changes, integrate genomic and proteomic data for a comprehensive understanding, investigate the effects of environmental stressors on the species, and conduct comparative studies with related species to identify unique adaptations. Also, location and sex need to be considered, and blood draws should be conducted at different times throughout the year in order to capture potential seasonal variations. It is especially critical to perform blood draws in rescue centers before administering treatments like deworming or ectoparasite treatment. Blood should be drawn during the initial veterinary examination, and then again after one month while the hedgehog is there and stabilized. Establishing RIs for wild animals is particularly challenging due to factors such as sex, age, and seasonality, requiring data on factors like age group (infant, juvenile, adult), sex, reproductive activity, and hibernation state, among others. These directions aim to enhance the knowledge of the hedgehog’s physiology, health, and ecological resilience. The holistic integration of environmental, geographic, anthropogenic, epidemiological, and potential zoonotic agent data is crucial to the identification of threats that endanger this species and humans. In this sense, establishing RIs for hematological and serum biochemical parameters is definitely a crucial pillar of the One Health concept.

## Figures and Tables

**Table 1:** Bacteria, viruses, protozoans, and fungi occurring in *Erinaceus europaeus* [1,12,15,16,17,18].

	Genus/Species	Symptoms **
Bacteria	*Salmonella* spp.	Anorexia, diarrhea, weight loss
	*Salmonella enteritidis*	
	*Salmonella typhimurium*	
	*Yersinia pseudotuberculosis*	Gastroenteritis, weight loss
	*Escherichia coli*	Not known
	*Bordetella bronchiseptica*	Tracheitis, catarrhal rhinitis, nasal discharge, broncho-pneumonia
	*Pasteurella multocida*	Secondary infections
	*Leptospira* spp.	Seldom appearance of clinical signs of infection
	*Anaplasma phagocytophilum*	Not known
	*Borrelia* spp.	Not known
	*Coxiella burnetti*	Not known
Viruses	TBE (tick-borne encephalitis)-Virus	Not known
	Mouth-and-Foot Disease	Vesicular lesions, day activeness
	Herpes virus	Not known
	MERS-CoV	Not known
	SARS-CoV-2	Not known
Fungi	*Trichophyton mentagrophytes* var*. erinacei*	Loss of spines and hair, crusty malformations of ear margins, lesions
Protozoa	*Isospora* spp.	Poor appetite, emaciation, lethargy, hemorrhagic diarrhea
	*Toxoplasma* spp.	Not known
	*Cryptosporidia* spp.	Not known

**—*Erinaceus europaeus* can be asymptomatic carriers and therefore not present any symptoms of disease.

**Table 2:** Description of the reference intervals of hematological parameters in the species *Erinaceus europaeus*, obtained from published studies [25,26,28].

Parameters	N	Mean ± SD	Median	Min–Max	RI	LRL 90% CI	URL 90% CI
RBC (M/μL)	50 ^a^	8.1 × 10^6 a^			6.2–10.0 ^a^		
	37 ^b^	7.10 ± 1.50 ^b^	7.20 ^b^	4.07–11.01 ^b^	4.1–10.2 ^b^	3.5–4.8 ^b^	9.5–10.9 ^b^
	34 ^c^	8.1 ± 1.1 × 10^6 c^	8.2 × 10^6 c^	5.6–10.2 × 10^6 c^	6.0–10.4 × 10^6 c^	5.4–6.5 × 10^6 c^	9.8–10.9 ^c^
%HCT	50 ^a^	33 ^a^			25–46 ^a^		
	37 ^b^	30.20 ± 5.30 ^b^	29.40 ^b^	17.30–41.40 ^b^	19.4–41.1 ^b^	16.7–21.9 ^b^	38.6–43.6 ^b^
	34 ^c^	32 ± 4 ^c^	33 ^c^	24–39 ^c^	25–41 ^c^	0.23–0.27 ^c^	0.39–0.42 ^c^
HGB (g/L)	50 ^a^	125 ^a^			96–166 ^a^		
	37 ^b^	10.20 ± 1.80 ^b^	10.00 ^b^	5.70–13.90 ^b^	6.6–13.8 ^b^	5.7–7.5 ^b^	13.0–14.6 ^b^
	34 ^c^	11.56 ± 1.61 ^c^	11.65 ^c^	8.30–14.60 ^c^	8.37–14.93 ^c^	7.61–9.23 ^c^	141.8–156.3 ^c^
MCV (fL)	50 ^a^	40.6 ^a^			6.2–10.0 ^a^		
	37 ^b^	42.90 ± 5.40 ^b^	40.90 ^b^	35.50–57.50 ^b^	34.3–55.8 ^b^	33.7–35.2 ^b^	50.5–59.7 ^b^
	34 ^c^	0.04 ± 0.0027 ^c^	0.0399 ^c^	0.0347–0.0458 ^c^	0.0345–0.0453 ^c^	0.0335–0.0358 ^c^	44.0–46.6 ^c^
MCH (pg)	50 ^a^	15.4 ^a^			13.7–17.5 ^a^		
	37 ^b^	14.40 ± 1.70 ^b^	14.00 ^b^	11.70–19.20 ^b^	12.0–19.1 ^b^	11.8–12.3 ^b^	17.4–20.9 ^b^
	34 ^c^	14.2 ± 0.7 ^c^	14.3 ^c^	12.5–15.8 ^c^	12.7–15.8 ^c^	12.4–13.1 ^c^	15.4–16.1 ^c^
MCHC (g/dL)	50 ^a^	38.1 ^a^			34.8–42.2 ^a^		
	37 ^c^	33.60 ± 1.30 ^c^	33.60 ^c^	31.40–36.70 ^c^	30.9–36.4 ^c^	30.2–31.6 ^c^	35.8–37.0 ^c^
	34 ^c^	35.70 ± 2.07 ^c^	35.65 ^c^	29.90–40.10 ^c^	31.29–40.0 ^c^	30.20–32.38 ^c^	385.8–412.0 ^c^
%RDW	37 ^b^	29.00 ± 3.20 ^b^	28.90 ^b^	24.20–37.80 ^b^	22.5–35.6 ^b^	21.0–24.1 ^b^	34.0–37.1 ^b^
%RETIC	37 ^b^	3.60 ± 3.40 ^b^	2.40 ^b^	0.20–14.00 ^b^	* ^b^	* ^b^	* ^b^
	34 ^c^	1.8 ± 1.3 ^c^	1.5 ^c^	0.2–5.2 ^c^	0.2–5.0 ^c^	0.0–0.3 ^c^	3.8–6.3 ^c^
RETIC (K/μL)	37 ^b^	222.70 ± 168.30 ^b^	183.10 ^b^	10.60–645.30 ^b^	11.5–705.7 ^b^	2.6–29.0 ^b^	549.2–892.2 ^b^
	34 ^c^	144.6 ± 111.2 ^c^	122.1 ^c^	15–438.7 ^c^	10.7–423.8 ^c^	3.2–23.4 ^c^	318.4–539.6 ^c^
%nRBCs	32 ^c^	2.2 ± 1.7 ^c^	1.8 ^c^	0.0–6.0 ^c^	0.1–6.5 ^c^	0.0–0.3 ^c^	4.4–8.6 ^c^
nRBCs (10^6^/L)	32 ^c^	0.18 ± 0.15 ^c^	0.13 ^c^	0.00–0.76 ^c^	0.01–0.63 ^c^	0.00–0.03 ^c^	0.40–1.11 ^c^
WBC (K/μL)	50 ^a^	7.4 × 10^9 a^			1.7–11.4 × 10^9 a^		
	37 ^b^	9.10 ± 3.20 ^b^	8.60 ^b^	2.22–15.31 ^b^	2.5–15.7 ^b^	1.0–4.0 ^b^	14.2–17.3 ^b^
	34 ^c^	8.4 ± 2.9 × 10^9 c^	8.1 × 10^9 c^	3.0–14.7 × 10^9 c^	2.2–13.9 × 10^9 c^	1.1–3.5 × 10^9 c^	12.3–15.7 × 10^9 c^
%NEU	37 ^b^	52.70 ± 14.10 ^b^	54.50 ^b^	5.00–74.20 ^b^	23.8–81.6 ^b^	16.1–30.6 ^b^	74.4–88.6 ^b^
	32 ^c^	35.1 ± 9.0 ^c^	34.6 ^c^	19.7–57.3 ^c^	16.2–53.1 ^c^	11.6–20.6 ^c^	47.6–58.0 ^c^
%LYM	37 ^b^	37.20 ± 11.00 ^b^	36.20 ^b^	19.30–60.10 ^b^	14.6–59.9 ^b^	9.6–20.0 ^b^	54.5–65.1 ^b^
	32 ^c^	50.0 ± 9.4 ^c^	50.4 ^c^	29.1–69.3 ^c^	31.2–69.6 ^c^	26.7–36.4 ^c^	65.0–75.0 ^c^
%MONO	37 ^b^	7.90 ± 6.20 ^b^	6.70 ^b^	2.80–42.30 ^b^	3.5–24.4 ^b^	3.1–4.2 ^b^	14.3–44.9 ^b^
	32 ^c^	2.7 ± 1.6 ^c^	2.6 ^c^	0.0–6.3 ^c^	0.1–7.5 ^c^	0.0–7.6 ^c^	5.9–9.1 ^c^
%EOS	37 ^b^	1.80 ± 1.80 ^b^	1.00 ^b^	0.00–6.50 ^b^	2.4–26.9 ^b^	2.0–3.0 ^b^	14.7–75.2 ^b^
	31 ^c^	7.5 ± 4.9 ^c^	6.7 ^c^	0.0–20.9 ^c^	0.0–12.0 ^c^	0.0–0.0 ^c^	8.5–16.3 ^c^
%BASO	37 ^b^	0.30 ± 0.40 ^b^	0.20 ^b^	0.00–1.00 ^b^	* ^b^	* ^b^	* ^b^
	32 ^c^	3.5 ± 2.1 ^c^	3.3 ^c^	0.4–10.0 ^c^	0.4–8.7 ^c^	0.2–0.9 ^c^	7.2–10.6 ^c^
NEU (K/μL)	50 ^a^	2.97 ^a^			0.42–6.38 ^a^		
	37 ^b^	4.90 ± 2.30 ^b^	4.50 ^b^	0.11–9.89 ^b^	0.8–10 ^b^	0.2–1.5 ^b^	8.2–11.5 ^b^
	32 ^c^	2.7 ± 1.2 × 10^9 c^	2.5 × 10^9 c^	0.9–6.1 × 10^9 c^	0.9–5.9 × 10^9 c^	0.8–1.2 × 10^9 c^	4.8–7.2 ^c^
LYM (K/μL)	50 ^a^	3.75 ^a^			0.85–7.77 ^a^		
	37 ^b^	3.30 ± 1.40 ^b^	3.30 ^b^	1.15–6.28 ^b^	0.4–6.3 ^b^	0.0–0.9 ^b^	5.5–6.8 ^b^
	32 ^c^	4.0 ± 1.6 × 10^9 c^	3.9 × 10^9 c^	1.5–7.8 × 10^9 c^	0.6–7.2 × 10^9 c^	0.0–1.4 × 10^9 c^	6.2–8.2 ^c^
MONO (K/μL)	50 ^a^	0.16 ^a^			0.00–0.48 ^a^		
	37 ^b^	0.60 ± 0.30 ^b^	0.60 ^b^	0.22–1.40 ^b^	0.2–1.4 ^b^	0.2–0.3 ^b^	1.1–1.6 ^b^
	32 ^c^	0.2 ± 0.1 × 10^9 c^	0.2 ^c^	0.0–0.6 × 10^9 c^	0.0–0.6 × 10^9 c^	0.0–0.0^c^	0.5–0.7 ^c^
EOS (K/μL)	50 ^a^	0.44 ^a^			0.00–1.35 ^a^		
	37 ^b^	0.20 ± 0.20 ^b^	0.10 ^b^	0.00–0.48 ^b^	* ^b^	* ^b^	* ^b^
	31 ^c^	0.6 ± 0.0 × 10^9 c^	0.5 ^c^	0.0–2.4 × 10^9 c^	0.0–2.2 × 10^9 c^	0.0–0.9 × 10^9 c^	1.6–3.0 ^c^
BASO (K/μL)	50 ^a^	0.08 ^a^			0.00–0.28 ^a^		
	37 ^b^	0.00 ± 0.00 ^b^	0.00 ^b^	0.00–0.11 ^b^	* ^b^	* ^b^	* ^b^
	32 ^c^	0.3 ± 0.2 × 10^9 c^	0.3 ^c^	0.0–0.6 × 10^9 c^	0.0–0.7 × 10^9 c^	0.0–0.1 × 10^9 c^	0.6–0.8 ^c^
PLT (K/μL)	50 ^a^	134 ^a^			29–338 ^a^		
	37 ^b^	268.30 ± 129.90 ^b^	237.00 ^b^	6.00–620.00 ^b^	31.3–567.1 ^b^	3.2–71.5 ^b^	472.2–660.3 ^b^
	34 ^c^	230.7 ± 102.6 × 10^9 c^	224.4 × 10^9 c^	48.0–462.0 × 10^9 c^	48.9–169.4 × 10^9 c^	12.4–86.1 × 10^9 c^	403.1–523.7 ^c^
MPV (fL)	33 ^b^	15.60 ± 1.10 ^b^	15.50 ^b^	12.40–17.80 ^b^	13.2–17.9 ^b^	12.7–13.9 ^b^	17.4–18.4 ^b^
%PCT	33 ^b^	0.40 ± 0.20 ^b^	0.40 ^b^	0.05–0.91 ^b^	0.1–0.8 ^b^	0.0–0.2 ^b^	0.7–0.9 ^b^

^a^—study of Lewis et al., 2002 [25]; ^b^—study of Rosa et al., 2023 [26]; ^c^—study of Rossi et al., 2014 [28]; N, Number of animals; SD, Standard Deviation; Min, Minimum; Max, Maximum; RI, Reference Interval; LRL, Lower Reference Limit; URL, Upper Reference Limit; CI, Confidence Interval. * Non-Computable. RBC, Red Blood Cells; HCT, Hematocrit; HGB, Hemoglobin; MCV, Mean Cell Volume; MCH, Mean Corpuscular Hemoglobin; MCHC, Mean Corpuscular Hemoglobin Concentration; RDW, Red Blood Cell Distribution Width; %RETIC, Reticulocyte percent; RETIC, Reticulocyte count; nRBCs, Nucleated Red Blood Cells count; %nRBCs, Nucleated Red Blood Cells percent; WBC, White Blood Cell; %NEU, Neutrophil percent; %LYM, Lymphocyte percent; %MONO, Monocyte percent; %EOS, Eosinophil percent; %BASO, Basophil percent; NEU, Neutrophil count; LYM, Lymphocyte count; MONO, Monocyte count; EOS, Eosinophil count; BASO, Basophil count; PLT, Platelet count; MPV, Mean Platelet Volume; PCT, Plateletcrit.

**Table 3:** Description of the reference intervals of biochemical parameters in the species *Erinaceus europaeus*, obtained from published studies [26,28].

Parameters	N	Mean ± SD	Median	Min–Max	RI	LRL 90% CI	URL 90% CI
GLUCOSE (mg/dL)	21 ^a^	108.50 ± 24.10 ^a^	109.00 ^a^	44.20–141.91 ^a^	57.0–160.0 ^a^	43.0–72.2 ^a^	144.0–175.3 ^a^
	30 ^b^	106.2 ± 14.4 ^b^	106.2 ^b^	77.4–131.4 ^b^	77.4–135.0 ^b^	126.0–84.6 ^b^	126.0–140.4 ^b^
TOTAL PROTEINS (g/dL)	34 ^a^	5.80 ± 1.20 ^a^	5.90 ^a^	3.55–8.59 ^a^	3.4–8.3 ^a^	2.8–3.9 ^a^	7.6–8.9 ^a^
	30 ^b^	6.88 ± 1.02 ^b^	6.74 ^b^	5.41–10.05 ^b^	4.44–8.83 ^b^	3.93–5.29 ^b^	8.15–9.59 ^b^
ALBUMIN (g/dL)	33 ^a^	3.30 ± 0.60 ^a^	3.20 ^a^	2.29–4.46 ^a^	2.1–4.5 ^a^	1.8–2.4 ^a^	4.2–4.8 ^a^
	30 ^b^	3.50 ± 0.34 ^b^	3.47 ^b^	3.03–4.57 ^b^	2.73–4.17 ^b^	2.51–2.94 ^b^	8.15–9.59 ^b^
ALT (U/L)	33 ^a^	129.20 ± 61.20 ^a^	125.00 ^a^	36.40–298.00 ^a^	* ^a^	* ^a^	* ^a^
	29 ^b^	81.0 ± 37.5 ^b^	78.0 ^b^	46.0–174.0 ^b^	43.3–194.3 ^b^	39.4–49.5 ^b^	153.9–247.0 ^b^
ALP (U/L)	34 ^a^	74.80 ± 48.00 ^a^	59.50 ^a^	18.00–203.10 ^a^	19.2–217.8 ^a^	17.8–22.7 ^a^	166.3–281.4 ^a^
	29 ^b^	141.4 ± 48.0 ^b^	135.2 ^b^	77.0–253.0 ^b^	68.3–255.7 ^b^	60.6–82.2 ^b^	215.8–292.6 ^b^
CREATININE (mg/dL)	33 ^a^	0.40 ± 0.20 ^a^	0.30 ^a^	0.10–0.77 ^a^	0.1–0.9 ^a^	0.1–0.1 ^a^	0.7–1.0 ^a^
	27 ^b^	0.65 ± 0.41 ^b^	0.55 ^b^	0.23–2.12 ^b^	0.23–1.98 ^b^	0.19–0.29 ^b^	1.33–3.01 ^b^
UREA (mg/dL)	23 ^a^	76.50 ± 42.70 ^a^	68.00 ^a^	16.20–215.40 ^a^	* ^a^	* ^a^	* ^a^
	30 ^b^	264.6 ± 55.8 ^b^	257.4 ^b^	181.8–417.6 ^b^	142.2–372.6 ^b^	113.4–180 ^b^	18.8–22.7 ^b^
PHOSPHORUS (mg/dL)	33 ^a^	7.70 ± 2.40 ^a^	7.40 ^a^	4.29–15.01 ^a^	2.7–12.7 ^a^	1.6–3.9 ^a^	11.4–14.0 ^a^
	26 ^b^	39.6 ± 9.0 ^b^	37.8 ^b^	27.0–63 ^b^	27.0–63.0 ^b^	25.2–28.8 ^b^	54.0–73.8 ^b^
CALCIUM (mg/dL)	33 ^a^	9.40 ± 1.60 ^a^	9.90 ^a^	2.55–11.35 ^a^	5.6–11.5 ^a^	1.4–7.4 ^a^	11.0–11.7 ^a^
	30 ^b^	30.6 ± 12.6 ^b^	32.4 ^b^	19.8–59.4 ^b^	23.4–57.6 ^b^	14.4–30.6 ^b^	52.2–59.4 ^b^
CHOLESTEROL (mg/dL)	15 ^a^	134.90 ± 50.00 ^a^	132.00 ^a^	24.00–224.00 ^a^	* ^a^	* ^a^	* ^a^
	30 ^b^	66.6 ± 14.4 ^b^	63.00 ^b^	43.2–95.4 ^b^	36.0–95.4 ^b^	28.8–43.2 ^b^	86.4–102.6 ^b^
TRIGLYCERIDES (mg/dL)	14 ^a^	55.70 ± 14.70 ^a^	56.00 ^a^	18.00–79.00 ^a^	22.9–88.5 ^a^	10.6–35.7 ^a^	75.1–100.9 ^a^
	29 ^b^	6.48 ± 2.70 ^b^	6.12 ^b^	1.98–12.96 ^b^	2.16–13.5 ^b^	1.62–3.06 ^b^	11.16–16.38 ^b^
GAMMA-GT (U/L)	14 ^a^	19.80 ± 17.90 ^a^	8.70 ^a^	2.60–56.00 ^a^	1.8–151.8 ^a^	* ^a^	* ^a^
	30 ^b^	17.7 ± 9.6 ^b^	16.6 ^b^	6.8–41.9 ^b^	5.2–47.5 ^b^	4.1–6.9 ^b^	38.0–64.2 ^b^
GLOBULINS (g/dL)	23 ^a^	2.30 ± 1.00 ^a^	2.10 ^a^	0.00–3.97 ^a^	0.4–4.7 ^a^	0.1–0.9 ^a^	3.6–5.9 ^a^
	30 ^b^	3.38 ± 0.76 ^b^	3.27 ^b^	2.37–5.48 ^b^	1.71–4.83 ^b^	1.33–2.17 ^b^	4.32–5.37 ^b^
AST (U/L)	9 ^a^	19.60 ± 5.90 ^a^	19.00 ^a^	13.90–30.70 ^a^	10.8–38.7 ^a^	9.6–13.4 ^a^	27.0–53.0 ^a^
BILIRUBIN (mg/dL)	10 ^a^	0.00 ± 0.00 ^a^	0.00 ^a^	0.01–0.03 ^a^	* ^a^	* ^a^	* ^a^
SODIUM (mmol/L)	20	142.90 ± 5.50 ^a^	142.70 ^a^	127.00–152.00 ^a^	129.8–153.1 ^a^	124.1–135.1 ^a^	150.3–155.6 ^a^
POTASSIUM (mmol/L)	20 ^a^	3.90 ± 0.70 ^a^	3.80 ^a^	2.15–5.44 ^a^	2.5–5.4 ^a^	2.0–2.9 ^a^	4.9–5.9 ^a^
CHLORIDE (mmol/L)	20 ^a^	110.20 ± 4.60 ^a^	109.40 ^a^	104.00–123.40 ^a^	100.3–120.1 ^a^	97.2–103.6 ^a^	117.0–123.2 ^a^
	Protein electrophoresis ^a^						
Albumin (g/dL)	12	2.60 ± 0.40	2.50	1.91–3.14	1.7–3.5	1.4–2.1	3.1–3.8
α1-Globulin (g/dL)	12	0.60 ± 0.10	0.60	0.42–0.94	0.3–0.9	0.2–0.4	0.8–1.1
α2-Globulin (g/dL)	12	0.60 ± 0.10	0.60	0.32–0.76	0.2–0.9	0.1–0.4	0.8–1.0
β-Globulin (g/dL)	12	1.20 ± 0.50	1.10	0.70–2.37	0.6–3.4	0.5–0.8	1.8–8.9
γ-Globulin (g/dL)	12	0.20 ± 0.10	0.10	0.03–0.49	0.0–0.1	0.0–0.0	0.4–2.5

^a^—study of Rosa et al., 2023 [26]; ^b^—study of Rossi et al., 2014 [28]; N, Number of animals; SD, Standard Deviation; Min, Minimum; Max, Maximum; RI, Reference Interval; LRL, Lower Reference Limit; URL, Upper Reference Limit; CI, Confidence Interval. * Non-computable. ALT, Alanine Aminotransferase; ALP, Alkaline Phosphatase; AST, Aspartate Aminotransferase.

## Data Availability

No new data were created or analyzed in this study. Data sharing is not applicable to this article.
